# Ambient atmospheric PM worsens mouse lung injury induced by influenza A virus through lysosomal dysfunction

**DOI:** 10.1186/s12931-023-02618-9

**Published:** 2023-12-06

**Authors:** Shunwang Li, Xiangwu Ju, Qiang Liu, Yiwu Yan, Cong Zhang, Yuhao Qin, Xingyu Deng, Chang Li, Mingyao Tian, Yanli Zhang, Ningyi Jin, Chengyu Jiang

**Affiliations:** 1 State Key Laboratory of Common Mechanism Research for Major Diseases, School of Basic Medicine Peking Union Medical College, Institute of Basic Medical Sciences Chinese Academy of Medical Sciences, Beijing, 100005 China; 2 Changchun Veterinary Research Institute, Chinese Academy of Agricultural Sciences, Changchun, 130122 China; 3 Center of Environmental and Health Sciences, Chinese Academy of Medical Sciences, Peking Union Medical College, Beijing, 100005 China

**Keywords:** Particulate matter, Influenza a virus, Lung injury

## Abstract

**Background:**

Particulate matter (PM) air pollution poses a significant risk to respiratory health and is especially linked with various infectious respiratory diseases such as influenza. Our previous studies have shown that H5N1 virus infection could induce alveolar epithelial A549 cell death by enhancing lysosomal dysfunction. This study aims to investigate the mechanisms underlying the effects of PM on influenza virus infections, with a particular focus on lysosomal dysfunction.

**Results:**

Here, we showed that PM nanoparticles such as silica and alumina could induce A549 cell death and lysosomal dysfunction, and degradation of lysosomal-associated membrane proteins (LAMPs), which are the most abundant lysosomal membrane proteins. The knockdown of LAMPs with siRNA facilitated cellular entry of both H1N1 and H5N1 influenza viruses. Furthermore, we demonstrated that silica and alumina synergistically increased alveolar epithelial cell death induced by H1N1 and H5N1 influenza viruses by enhancing lysosomal dysfunction via LAMP degradation and promoting viral entry. In vivo, lung injury in the H5N1 virus infection-induced model was exacerbated by pre-exposure to silica, resulting in an increase in the wet/dry ratio and histopathological score.

**Conclusions:**

Our findings reveal the mechanism underlying the synergistic effect of nanoparticles in the early stage of the influenza virus life cycle and may explain the increased number of respiratory patients during periods of air pollution.

**Supplementary Information:**

The online version contains supplementary material available at 10.1186/s12931-023-02618-9.

## Background

Air pollution is one of the world’s leading risk factors for human morbidity and mortality worldwide, especially in individuals with pre-existing lung diseases [1–5]. Particulate matter (PM) in polluted air plays a critical role in changing air quality, human health, and the climate. Silica, alumina, and iron oxide are present at high percentages in ambient atmospheric PM and can be deposited and retained in different regions of the human airway [1, 6]. In particular, silica and alumina have been found to be significantly increased in the hilar lymph nodes in autopsied patients with idiopathic pulmonary fibrosis (IPF) [5]. Furthermore, fine nanoscale PM can more easily penetrate into the lungs and cause respiratory diseases, and it is thus a major health risk factor of air pollution.

Various infectious respiratory diseases, such as influenza, have been clearly linked to air pollution [7–9]. Influenza is a contagious respiratory disease caused mostly by infection of the respiratory tract with influenza A virus. Influenza can cause mild to severe illness and can lead to serious outcomes resulting in hospitalization or even death, constituting a major global public health problem. Influenza prevalence peaks in winter, and hospital admissions of patients with influenza virus infection have been reported to increase during periods of heavy pollution [9–12]. However, the mechanisms underlying the potential association between the toxicological effects of PM and influenza A virus infection remain unclear, and further studies are urgently needed.

Lysosomes contain a variety of hydrolytic enzymes and primarily degrade endocytosed macromolecules or infected pathogens, functioning as recycling centers. Endocytosis of nanoparticles often ends with lysosomal internalization, and the realization that lysosomal dysfunction is an important mechanism of nanoparticle toxicity is increasing [13]. Although most invading viruses are lysed and degraded in the lysosome, some viruses have evolved the ability to survive this toxic acidic environment [14]. Indeed, previous reports have shown that HIV, adenovirus, and poliovirus can cause lysosomal dysfunction [15]. In particular, our previous study showed that neuraminidase (NA) of influenza virus may bind directly to lysosomal-associated membrane proteins (LAMPs) in lysosomes, reducing their glycosylation, and disrupting lysosomal integrity [16]. Thus, lysosomal dysfunction may be induced by different causes, including nanoparticles and influenza virus infection.

The epidemiological survey and previous studies brought us a new hypothesis that PM, especially nanoparticles in air, might weaken the ability of lysosomes to destroy viruses and synergize with viruses to facilitate viral entry and replication [7–9, 15, 16]. Then, we designed experiments to analyze the combined effects of nanoparticles in polluted air and influenza virus during the attack on human lungs in vitro and in vivo and tried to explain why the observed pollutants were significantly associated with respiratory mortality, particularly influenza. Our findings show that nanoparticles such as silica, could synergize with influenza virus infection by promoting both viral entry and lysosomal escape, thus increasing cell death in vitro. Accordingly, our in vivo results showed that lung injury induced by influenza virus was exacerbated when the mice were pre-exposed to nanoscale silica. Lysosomal dysfunction may be the hub in the attack pathways of nanoparticles and influenza viruses.

## Materials and methods

### Preparation of nanoparticles and solutions

The nanoparticle powders silicon dioxide (10–20 nm, 637,238), aluminum oxide (~ 13 nm, 718,475) and iron(II, III) oxide (< 50 nm) (637,106) were purchased from Sigma-Aldrich (St. Louis, MO). For in vitro experiments, the PM samples were suspended in PBS and prepared in 10 mg/mL stock solutions. For in vivo experiments, the PM samples were suspended in saline to a stock solution concentration of 50 mg/mL. Then, all working solutions were mixed by ultrasonication for 15 min. The PMs suspensions were rolled for 10 s to ensure uniformity before each use and were then diluted to a working concentration of 30 μg/mL.

### Influenza viruses

The seasonal influenza viruses H1N1 (A/New Caledonia/20/1999 (H1N1)) and avian influenza virus H5N1 (A/Jilin/9/2004 (H5N1)) were used in this study. Experiments with live influenza viruses were performed in biosafety level 3 facilities in accordance with governmental and institutional guidelines. Viruses were propagated and titered as described previously [17]. In brief, viruses were propagated by inoculation into 10- to 11-day-old, specific pathogen-free (SPF) embryonated chicken eggs and titered using the Reed-Muench method with MDCK cells. The titers are expressed as the median tissue culture infectious dose (TCID50) per milliliter of supernatant. Unless otherwise stated, a multiplicity of infection (M.O.I.) of 4 was used for H1N1 or H5N1 influenza virus infection in this study.

### In vivo experiments

Wild-type C57BL/6 mice (6 to 8 weeks old; catalog no. 5,653,791, RRID MGI: 5,653,791) were purchased from Vital River (Beijing, China). Mice were first intratracheally injected with silica nanoparticles, and mice in the control group were administered allantoic fluid (AF). After 48 h, mice were intratracheally instilled with live H5N1 virus (10^6^ TCID50). Mice were sacrificed 3 days after virus infection, and bilateral lungs were collected to assess lung injury and pulmonary edema. The animal experiments in this work were approved by the Ethics Committee of the Institute of Basic Medical Sciences, Chinese Academy of Medical Sciences (ACUC-A02-2017-014), and adhered to the Chinese National Guidelines for the Care and Use of Laboratory Animals and the institutional animal care guidelines.

### Antibodies and reagents

Antibodies against LAMP1 (3243) (for immunoblotting) were purchased from Cell Signaling Technology (Danvers, MA, USA). Antibodies against β-actin (clone AC-15, A5441) were purchased from Sigma-Aldrich (St. Louis, Missouri, USA). Antibodies against LAMP2 (clone H4B4, sc-18,822) were purchased from Santa Cruz Biotechnology (Santa Cruz, CA, USA). The anti-NP (C01321M) antibody was purchased from Millipore (Billerica, MA, USA), and horseradish peroxidase (HRP)-conjugated secondary antibodies were purchased from Santa Cruz Biotechnology. 2,2,2-Tribromoethanol (T48402) was purchased from Sigma-Aldrich.

Dulbecco’s modified essential medium (DMEM), fetal calf serum (FCS), and antibiotics were obtained from Gibco (Life Technologies) (Carlsbad, CA, USA). F-12/Ham’s nutrient medium and Halt Protease Inhibitor Cocktail were obtained from Thermo Fisher Scientific (Waltham, MA, USA), and 3-(4,5-dimethylthiazol-2-yl)-5-(3-carboxymethoxyphenyl)-2-(4-sulfophenyl)-2 H-tetrazolium inner salt (MTS) was procured from Promega Corporation (Madison, WI, USA). Acridine orange (AO) was acquired from Merck (Billerica, MA, USA). LysoTracker® Red DND-99 and ProLong® Gold Antifade Mountant with DAPI were purchased from Molecular Probes (Life Technologies) (Eugene, Oregon, USA).

### Cell culture

The human lung adenocarcinoma A549 cell line was purchased from ATCC (Rockville, MA, USA) and cultured in F-12/Ham’s medium (HyClone) supplemented with 10% fetal bovine serum (FBS) (Gibco), 100 units/mL penicillin, and 100 units/mL streptomycin at 37 °C in 5% CO_2_.

### Immunoblot analysis

Immunoblot analysis was performed as described previously [18]. Cells were collected and lysed with RIPA lysis buffer (50 mM Tris-HCl [pH 7.5], 150 mM NaCl, 1% NP-40, 0.1% sodium dodecyl sulfate [SDS], 5 mM EDTA, 0.5% sodium deoxycholate, 1 mM Na_3_VO_4_, 1 mM NaF, and protease inhibitor cocktails). The total protein concentration of each sample was determined with a BCA protein assay kit (TianGen, Beijing, China), and samples were boiled with 2× loading buffer after equalization of the protein concentration. Samples were resolved by 10% or 12% SDS-polyacrylamide gel electrophoresis (SDS-PAGE), and proteins were transferred onto nitrocellulose filter membranes. Membranes were blocked with 2% albumin and were then incubated with the appropriate primary antibodies overnight prior to incubation with HRP-conjugated secondary antibodies at room temperature (RT) for 1 h. Binding of secondary antibodies was detected using a Kodak film exposure detection system, and the film was scanned and analyzed. For detection of a second primary antibody, nitrocellulose membranes were stripped with stripping buffer (1% SDS, 25 mM glycine [pH 2.0]) and were then incubated with another primary antibody.

### Real-time quantitative PCR (q-PCR) analysis

Cells were lysed with TRIzol reagent (Invitrogen), and total RNA was isolated following the standard protocol provided by the manufacturer. Complementary DNA (cDNA) was synthesized from 1.5 μg of total RNA with a High-Capacity cDNA Reverse Transcription Kit (Applied Biosystems, Life Technologies). PCR amplification was performed with FastStart Universal SYBR Green Master Mix and Rox (Roche) in a LightCycler 480 thermal cycler. The relative gene expression levels were calculated using the C_t_ values and were normalized to the expression levels of human glyceraldehyde-3-phosphate dehydrogenase (GAPDH), the reference gene. The specific primers used were as follows: GAPDH forward: 5’-GGTGGTCTCCTCTGACTTCAACA-3’, GAPDH reverse: 5’-GTTGCTGTAGCCAAATTCGTTGT-3’; M1 forward: 5’- CTCTCTATCATCCCGTCAG-3’, M1 reverse: 5’-GTCTTGTCTTTAGCCATTCC-3’; M2 forward: 5’-ATTGTGGATTCTTGATCGTC-3’, M2 reverse: 5’-TGACAAAATGACCATCGTC-3’.

### siRNA transfection

All siRNAs used in this study were designed and synthesized by RiboBio (Guangzhou, China). Before siRNA transfection, A549 cells were seeded at 3 × 10^5^ cells/well in 12-well plates. Twenty-four hours later, cells were transfected with Lipofectamine RNAiMax reagent (Invitrogen) and the indicated siRNA (50 nM) diluted in Opti-MEM (Invitrogen) following the manufacturer’s guidelines. Forty-eight hours post siRNA transfection, downstream experiments were performed. The siRNA sequences for LAMP1 (siLAMP1) were 5’-CAAUGCGAGCUCCAAAGAAdTdT-3’/3’-dTdT GUUACGCUCGAGGUUUCUU-5’, and the siRNA sequences for LAMP2 (siLAMP2) were 5’- GCGGUCUUAUGCAUUGGAAdTdT-3’/3’-dTdT CGCCAGAAUACGUAACCUU-5’.

### Confocal microscopy

A549 cells were grown on coverslips in 24-well plates. At the indicated time points after virus infection, the culture supernatant was removed, and the coverslips were washed three times with PBS. Then, the cells were fixed with 4% paraformaldehyde for 10 min, permeabilized with 0.2% Triton X-100 for 5 min, and blocked with 10% goat serum for an additional 30 min. The coverslips were then incubated first with the indicated primary antibody at RT for 1 h and then with an Alexa Fluor 488- or Alexa Fluor 568-labeled secondary antibody (Molecular Probes). The subcellular localization of each target protein was observed using confocal laser scanning microscopy (FV-1000, Olympus, Tokyo, Japan), and the images were analyzed with Olympus Fluoview 3.0 software. For analysis of NP-positive nuclei, at least 64 images were continuously acquired automatically by the confocal microscope and were assessed with ImageJ software (National Institutes of Health, Bethesda, Maryland, USA).

To label cellular lysosomes, cells were incubated with 300 nM LysoTracker Red DND-99 (Molecular Probes) in fresh medium for 30 min at 37 °C and were then extensively washed with PBS and fixed with 4% paraformaldehyde for 15 min. Finally, the coverslips were mounted with ProLong® Gold Antifade Mountant with DAPI.

### Cell viability assays

A549 cells were infected with virus at an M.O.I. of 4 or with an equal volume of vehicle for the indicated durations. Cell viability was then determined by an MTS assay (Promega, Madison, WI, USA). In the treatment experiments, nanoparticles (30 μg/mL) were added 1 h before virus administration or at the indicated time points. The MTS assay was performed 48 h post virus infection.

### Nanoparticle and influenza virus instillation, lung wet/dry ratio assay and histopathological examination of mouse lung tissue

Mice were randomly grouped. After anesthesia was induced by an intraperitoneal injection of 2,2,2-tribromoethanol (500 mg/kg body weight), the tracheas of the mice were separated surgically. The nanoparticle suspensions were administered by intratracheal instillation (30 mg/kg body weight). Instillation was selected as the exposure method over other methods, such as inhalation and aspiration, because of the comparative accuracy of the nanoparticle intake during manipulation. Saline was used as the control. After 48 h, a second tracheal separation under anesthesia was performed, and H5N1 and H1N1 viruses (TCID_50_ = 10^6^, 4 μL/g body weight) were intratracheally instilled. AF was used as the control. Seventy-two hours post -influenza virus infection, mice were sacrificed. The lung tissues were rapidly collected for further examination. In the lung wet/dry ratio assay, which allows evaluation of pulmonary edema, the wet weight of the lungs was measured. To obtain the dry weight, the lungs were dried at 65 °C for 24 h. For histopathological examination, lungs were fixed in formalin for 48 h and embedded in paraffin, and 4-μm sections were stained with hematoxylin and eosin (H&E). Micrographs were acquired with a Leica DM3000 microscope connected to a Leica DFC 420 digital camera and Leica Qwin V3 software. The number of neutrophils per field of view (400×) was determined in 100 microscopic fields per group.

### Statistical analysis

All data are shown as the mean ± S.E.M. values. Measurements at single time points were analyzed by ANOVA and, if the differences were significant, the measurements were further analyzed by a two-tailed t-test. All statistical tests were conducted using GraphPad Prism 5.0 (GraphPad Software, San Diego, CA, USA). *P* < 0.05 indicates a statistically significant difference; *P* < 0.01, a highly significant difference (**).

## Results

### Silica and alumina nanoparticles induce lysosomal dysfunction

**Fig. 1:**
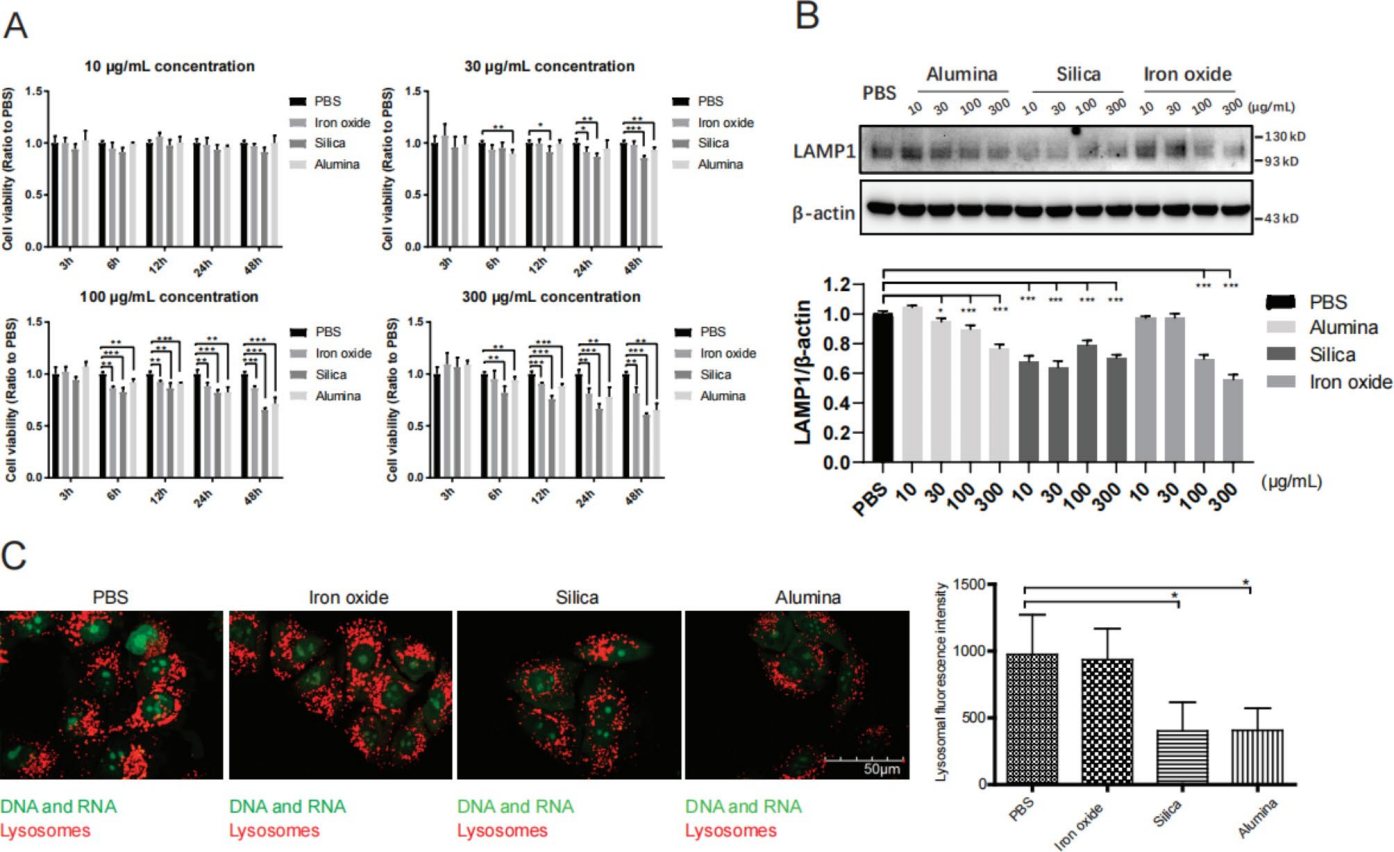
Lysosome dysfunction upon incubation with nanoparticles. (**A**) A549 cells were incubated with PBS, iron oxide, silica, or alumina for 48 h, and then their viability was examined by an MTS assay. (**B**) Immunoblot analysis of LAMP1 in A549 cells treated with nanoparticles at the indicated concentrations; β-actin was used as the control. (**C**) Twenty-four hours post incubation with 30 μg/mL nanoparticles, cells were stained with LysoTracker. Images were acquired using confocal microscopy, and the fluorescence signal intensity of each cell, as shown on the right, was estimated by examining more than 100 cells. The data are presented as the mean ± S.E.M. of three independent experiments. *P < 0.05, **P < 0.01

Nanoparticles are known to be sequestered within lysosomes, leading to potential lysosomal dysfunction. Previous reports have shown that numerous nanomaterials, such as fine silica and alumina particles, have been associated with lysosomal dysfunction [19]. As silica, alumina, and iron oxide are prevalent in polluted air [1, 4, 6], we examined the effects of these particles on cell survival and lysosomal function in A549 cells, a human alveolar type II cell line derived from lung adenoma, by MTS assays, LAMP immunoblotting and active lysosome labeling with LysoTracker. Our results showed that treatment with 30 μg/mL silica or alumina significantly decreased the cell survival rate at 48 h compared with that of the vehicle control- and iron oxide-treated cells (Fig. 1A). As the lysosomal membrane glycoproteins LAMP are important for maintaining the structural integrity of lysosomes [20], we investigated changes in these proteins in A549 cells treated with nanoparticles. We observed that LAMP1 was significantly degraded in A549 cells upon incubation for 24 h with silica and alumina at 30, 100, and 300 μg/mL (Fig. 1B). We then used confocal microscopy to examine the amounts and integrity of lysosomes. After 24 h of incubation with 30 μg/mL silica and alumina nanoparticles, the number of active lysosomes (labeled with LysoTracker) in A549 cells was significantly decreased compared with that in cells treated with the vehicle control and 30 μg/mL iron oxide (Fig. 1C). The measured staining intensity of LysoTracker, indicating active lysosomes, showed a decreased lysosome content in silica- and alumina-treated A549 cells, suggesting increased lysosomal dysfunction (Fig. 1C). Collectively, these results suggest that incubation with silica and alumina nanoparticles but not upon incubation with iron oxide could cause lysosomal dysfunction.

### Lysosomal dysfunction promotes the cell entry of influenza virus

**Fig. 2:**
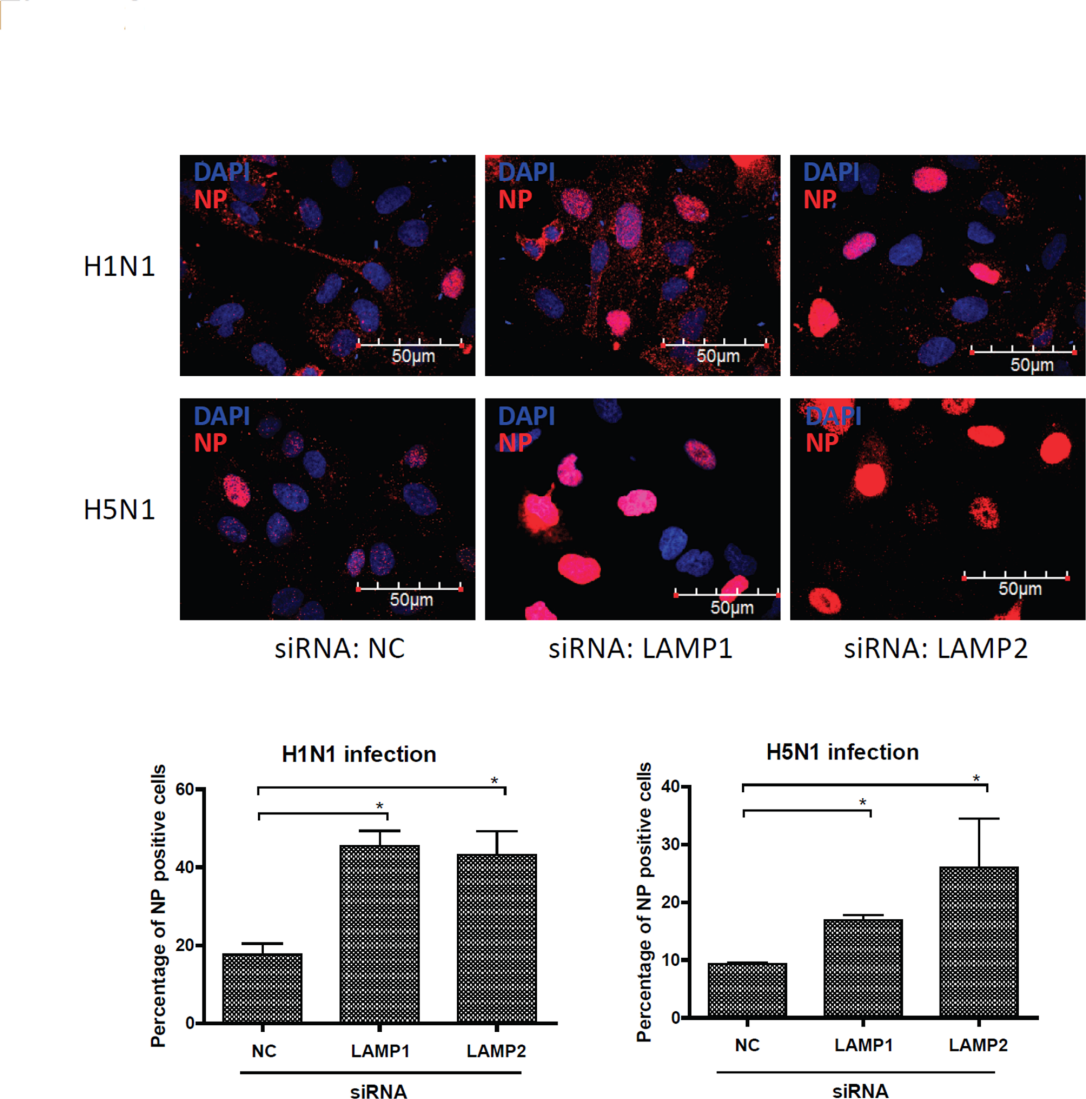
Lysosome dysfunction increases influenza virus entry. Confocal microscopy of influenza virus entry into A549 cells, which were transfected with a nontargeting control siRNA (NC) or a LAMP1- or LAMP2-specific siRNA for 48 h and then treated with vehicle, H1N1 virus, or H5N1 virus for 4 h. The graph in the lower panel indicates the percentage of NP-positive cells determined using ImageJ software from at least 1,000 cells. Scale bars, 50 μm. The data are shown as the mean and standard deviation values. *P < 0.05

Lysosomal impairment resulting from LAMP deficiency may cause lysosomal overload and reduce its ability to clear invading viruses. To analyze the effects of siLAMPs on influenza virus entry, we quantified the number of viral NP-positive nuclei 4 h post-infection (Fig. 2). Our results revealed that both siLAMP1 and siLAMP2 markedly promoted the entry of influenza virus into epithelial cells, as compared to siNC (Fig. 2). These findings suggest that lysosomal dysfunction induced by siLAMPs can enhance influenza virus entry into infected cells.

### Silica and alumina nanoparticles synergize with influenza viruses to exacerbate lysosomal dysfunction at the early stage of the viral life cycle

Taken together, the results from Figs. 1 and 2 indicate that nanoparticle-induced lysosomal dysfunction may share similarities with the effect of siLAMPs, potentially contributing to the reported association between influenza virus infection and air pollutants. Consequently, we conducted additional investigations to determine if lysosomal dysfunction induced by nanoparticles could enhance influenza virus entry.

**Fig. 3:**
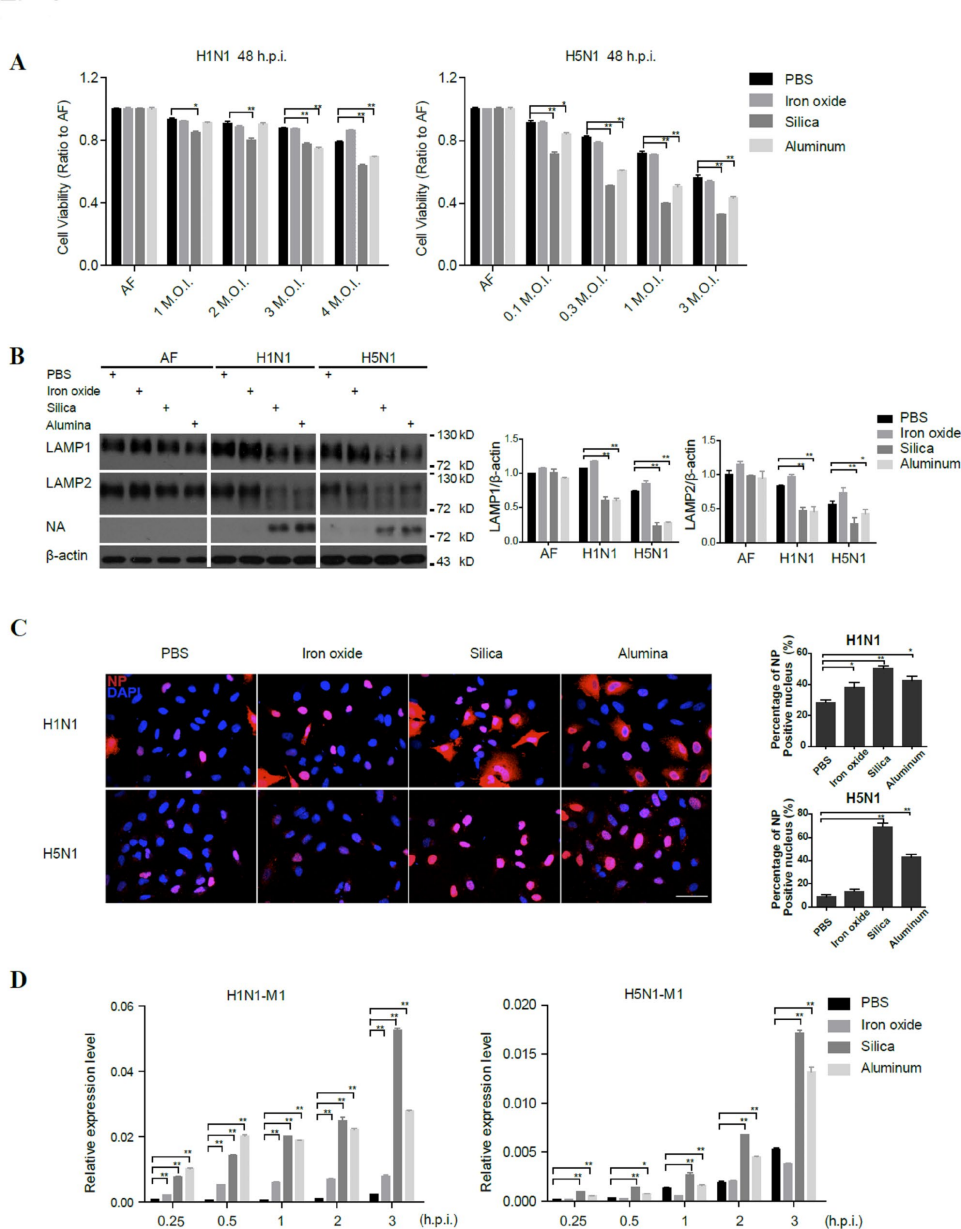
Silica and alumina oxide particles synergize with influenza viruses to aggravate lysosomal dysfunction. (**A**) MTS assay of evaluating the viability of A549 cells treated with PBS, iron oxide (100 μg/mL), silica oxide (100 μg/mL), or alumina (30 μg/mL) combined with vehicle or the indicated amounts of seasonal H1N1 or H5N1 virus 48 h post-infection. (**B**) Immunoblot analysis of LAMP1 and LAMP2 deglycosylation in A549 cells treated with vehicle, H1N1 (M.O.I., 3) or H5N1 (M.O.I., 0.3) virus combined with PBS, iron oxide (100 μg/mL), silica oxide (100 μg/mL), or alumina (30 μg/mL), separately, at 24 h post-infection. LAMP1 and LAMP2 levels were normalized to β-actin levels. (**C**) Confocal microscopy analysis of viral NP-positive nuclei in A549 cells treated with vehicle or H1N1 (M.O.I., 3) or H5N1 (M.O.I., 0.3) virus combined with PBS, iron oxide (100 μg/mL), silica oxide (100 μg/mL), or alumina (30 μg/mL) for 4 h. The graph on the right indicates the percentage of NP-positive cells determined using ImageJ software from at least 1,000 cells. Scale bars, 50 μm. (**D**) q-PCR detection of the influenza virus M1 gene in A549 cells infected with H1N1 (M.O.I., 3) or H5N1 (M.O.I., 0.3) virus combined with PBS, iron oxide (100 μg/mL), silica oxide (100 μg/mL), or alumina (30 μg/mL), separately, at 0.25 h, 0.5 h, 1 h, 2 h, 3 h after infection. The data are presented as the mean ± S.E.M. of three independent experiments. *P < 0.05, **P < 0.01

We first examined whether adding either of these nanoparticles to H1N1 or H5N1 infection could enhance alveolar epithelial cell death. Consistent with the lysosomal dysfunction data in Fig. 1, silica and alumina exacerbated the cell death induced by H1N1 and H5N1 virus infection, albeit to different extents, while iron oxide or vehicle PBS could not. (Fig. 3A and Fig. S1). Additionally, we observed increased deglycosylation of the LAMP1 and LAMP2 proteins in the groups with influenza virus infection combined with silica or alumina treatment (Fig. 3B), suggesting increased lysosomal dysfunction and decreased viral clearance.

Next, we examined the effects of these nanoparticles on the early stage of viral entry within 6 h post-virus infection and found that the entry of H1N1 or H5N1 influenza virus into epithelial cells was significantly enhanced by both silica and alumina nanoparticles, as determined by the number of NP-positive nuclei 4 h post virus infection (Fig. 3C). Furthermore, silica and alumina nanoparticles also increased viral loads shown by M1 and M2 at early and later infection stages (Fig. 3D and Fig. S2). These results suggest that nanoparticles may indeed enhance influenza virus entry during the early stage of the H1N1 and H5N1 influenza virus life cycle.

### Silica nanoparticles synergize with influenza viruses to exacerbate lung injury in mice

**Fig. 4:**
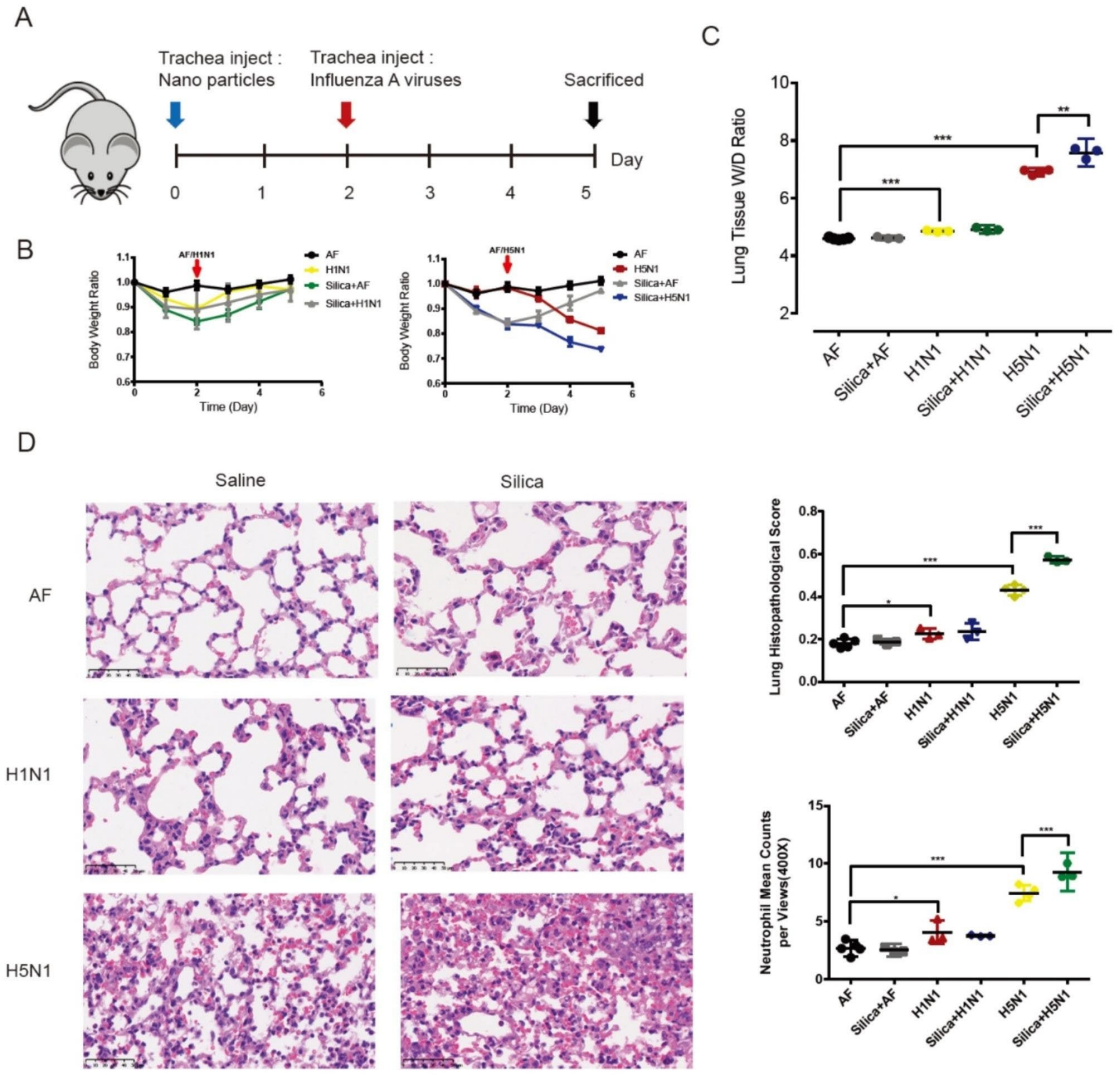
Silica synergizes with influenza viruses to exacerbate lung injury in mice. (**A**) Schematic diagram showing the synergistic instillation of nanoparticles and influenza viruses. Nanoparticles were injected at a dose of 30 mg/kg body weight. Mice were infected with H5N1 and H1N1 viruses (TCID_50_ = 10^6^) at a dose of 4 mL/kg body weight 2 days after nanoparticle injection. Mice were sacrificed, and lung tissues were harvested on day 5 (n = 3 to 5 per group). (**B**) Body weights were recorded each day during the nanoparticle and influenza virus synergistic exposure experiments. (**C**) Lung wet/dry ratio. (**D**) Representative images of H&E staining. The histopathological scores of lung injury and the numbers of infiltrating neutrophils per microscopic field (n = 30–40 fields per lung section) are shown in the right panel. Scale bars, 50 μm. The data are presented as the mean ± S.E.M. of three independent experiments. *, *P* < 0.05; **, *P* < 0.01;***, *P* < 0.001

We then mimicked this combinatorial attack by nanoparticles in air pollutants and influenza virus in vivo. C57BL mice were first intratracheally injected with silica nanoparticles and were then exposed to influenza virus 48 h later. The body weight continuously declined in the silica plus H5N1 virus group, while that in the silica plus H1N1 virus group was gradually restored 2 days post silica instillation (Fig. 4A and B). The degree of acute lung injury, as indicated by the lung wet/dry ratio after 72 h of exposure to influenza virus, was significantly increased in the group treated with both silica and H5N1 influenza virus compared with that in the groups infected with influenza virus or treated with silica instillation alone (Fig. 4C). HE staining results and the related pathological scores of mouse lung tissue also indicated worsening of lung injury in the combined-treatment group infected with H5N1 virus post silica instillation, compared with the single-treatment groups (Fig. 4D).

## Discussion

Many epidemiological studies have shown that air pollutants are significantly associated with respiratory mortality. However, these epidemiological studies did not reveal the related mechanisms. The putative mechanisms by which PM increases respiratory infection risk in the population co-exposed to pollution and respiratory pathogens have been suggested as follows in previous studies: impairment of airway immunity leading to increased susceptibility, impairment of the immune response and protracted symptoms leading to increased infectiousness, or exacerbation of inflammation and oxidative stress leading to more severe disease outcome [21]. These mechanisms are supported by a few studies investigating the mechanisms underlying the relationship between air pollution and influenza infection, most of which focused on immune defense mechanisms and oxidative stress in the lungs [22, 23]. One nanomaterial, single-walled carbon nanotubes (SWCNTs), was reported to modulate the immune system, suppress antiviral mechanisms in lung cells, impair mitochondrial function, and modulate viral receptors. These effects synergized to increase susceptibility to pandemic influenza A virus infection of lung epithelial cells [24].

In contrast, our study indicated that the effect of nanoparticles in PM on infectivity is mediated by lysosomal dysfunction pathways.

Our studies [15]and other [12] found that influenza virus and nanoparticles impaired lysosomal function, resulting in viral escape and nanoparticle toxicity, respectively [13, 16]. We further designed this study to provide in vitro and in vivo experimental data to determine whether nanoparticle-induced lysosomal dysfunction-related toxicity could synergize with influenza A virus in lysosomal dysfunction-related cell entry and viral replication.

Lysosomal degradation pathways play a vital role in cellular homeostasis, and lysosomal dysfunction has been associated with several disease states [13, 25]. Influenza virus enters cells through the endocytic pathway and is first packaged in endosomes. Then, endosomes fuse with lysosomes, where influenza viruses are degraded and cleared [26], with only a few escaping from the endosome. Different factors may cause lysosomal dysfunction, which further leads to blocked viral clearance, increased viral load, and enhanced cell death [27]. We previously reported that lysosomal dysfunction can be induced by deglycosylation of LAMP1 and LAMP2 after influenza virus infection [16]. Because LAMPS constitute approximately half of the total lysosomal membrane protein content and are heavily glycosylated, they play critical roles in lysosome function by preventing the release of hydrolases into the cytoplasm [28]. We thereby examined the effects of siLAMP on viral entry within 6 h post-virus infection, because the average time between the cellular entry of influenza virus and the production of progeny influenza virions is 6 h [29, 30]. Here, we further showed that deficiency of LAMPs induced by siRNA directly resulted in enhanced cell entry of influenza virus. This enhancement might result from impaired lysosomal function [31], which inhibits viral degradation and clearance [32]. A positive feedback loop may thus be formed during the infection process: infection leads to deglycosylation of LAMP1 and LAMP2 and subsequent lysosomal dysfunction, while lysosomal dysfunction further enhances viral entry into the cell.

Silica, alumina, and iron oxide have been found in polluted air as well as in human lungs. These components of ambient atmospheric PM can be deposited and retained at high particle concentrations in different regions of the human airway [1]. Among these components, silica is the largest species deposited in the lung, constituting almost half of the total particles in the large airways and one-quarter of those in the parenchyma [1]. Inhalation of silica can lead to acute lung inflammation, while chronic exposure causes pneumoconiosis silicosis. In addition, higher concentrations of silica and alumina were found in the hilar lymph nodes in patients with IPF than in controls without IPF; thus, these components are risk factors for human health [5]. Phagocytosis of silica by resident macrophages can elicit an inflammatory response featuring the release of IL-1β and TNFα. Moreover, silica and a wide variety of nanomaterials can induce lysosomal membrane permeabilization and have been associated with lysosomal dysfunction, which may be a common result of nanoparticle exposure since nanoparticles are generally sequestered in lysosomes [4, 33–35, 35]. In our study, we detected decreased LAMP1 levels and decreased fluorescence intensity using the fluorescent probe LysoTracker dyes in silica- and alumina-treated A549 cells (Fig. 1). The ingested LysoTracker is trafficked into the lysosomal pathway and stains acidic structures with high selectivity. Therefore, in addition to the decreased LAMP1 levels, the decreased fluorescence intensity indicates lysosomal rupture or the leakage of lysosomal contents into the cytosol in nanoparticle-treated cells. Nanoparticle-induced lysosomal dysfunction might be implicated in the observed increase in hospital admission of patients with influenza virus infection during periods of heavy pollution. Therefore, we further investigated whether silica, alumina, and iron oxide nanoparticles can enhance and synergize with influenza A virus to promote cell entry, which is closely related to the escape of influenza virus from inactivated lysosomes during its infection cycle.

As predicted, when combined with influenza viruses, the reported air pollution components silica and alumina enhanced influenza virus entry and increased the viral load; furthermore, more severe LAMP deglycosylation was observed in the group treated with nanoparticles and infected with the virus compared with the groups with either nanoparticle treatment alone or infection alone. Consistent with this finding, cell death was enhanced by the combination of influenza virus infection with silica or alumina nanoparticle treatment. In contrast, iron oxide did not induce a significant decrease in LAMP1 and LAMP2 immunostaining or LysoTracker labeling, consistent with the relatively small influence of iron oxide combined with influenza A virus infection on influenza A virus cell entry and viral load, especially in the H5N1 groups. This variability may be attributed to the distinct properties of each nanoparticle, including their capacity to penetrate the cell and their interactions with lysosomes.

Alveoli, composed of type 2 and type 1 alveolar epithelial cells, are the major components of the blood-oxygen barrier and act as the gas exchange sites in the lung. As the combination of silica treatment and H5N1 virus infection enhanced alveolar epithelial cell death, we accordingly found more severe lung edema and higher lung histopathological scores in the combination treatment group in our mouse lung injury model due to the damaged respiratory blood-oxygen barrier in vivo.

## Conclusions

**Fig. 5:**
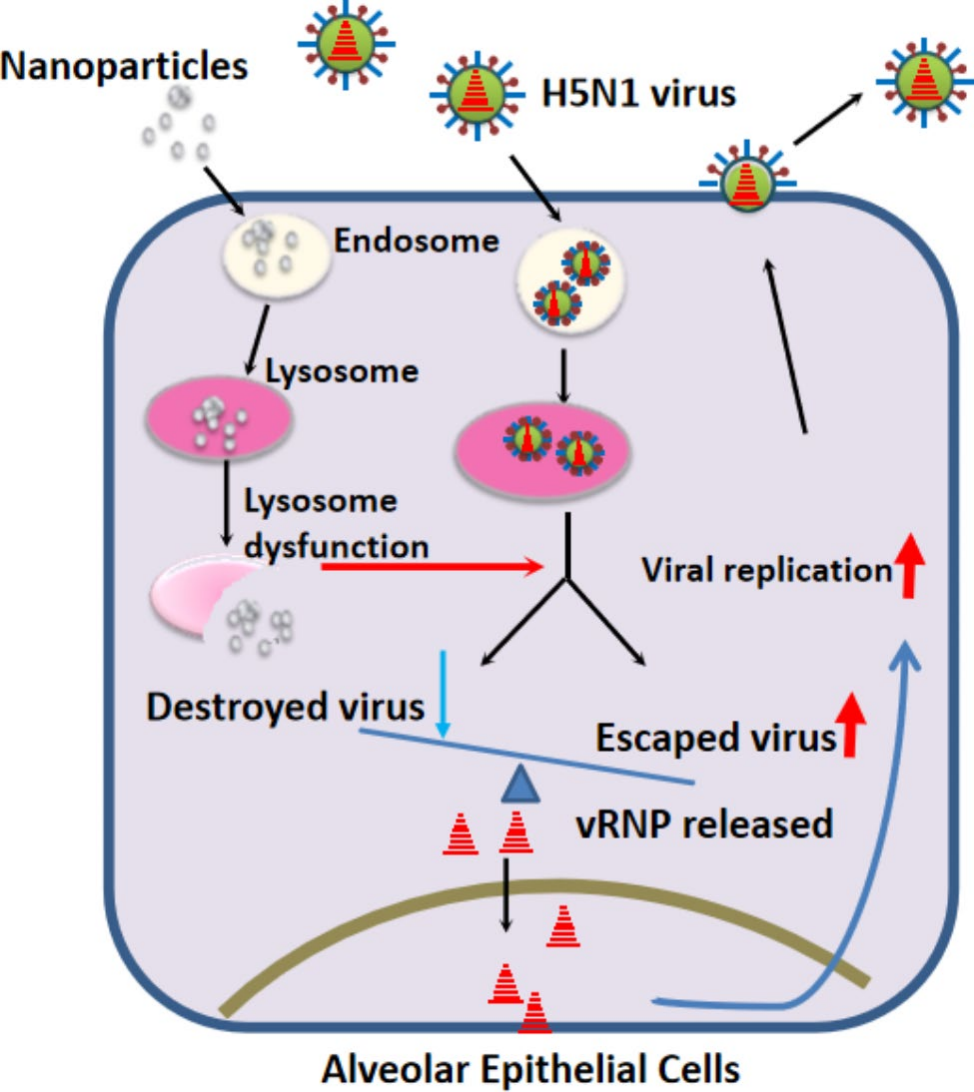
A proposed model for the mechanism that nanoparticles synergize with influenza virus infection

Our findings reveal the mechanism underlying the combinatorial effects of nanoparticles on the early stage of the influenza virus life cycle in vitro (Fig. 5) and may explain the increased number of respiratory patients during periods of air pollution. Our study may provide in vitro and in vivo evidence to explain why a significantly increased number of patients with influenza A virus infection are hospitalized in polluted weather, as observed in previous clinical investigations [11, 12].

### Electronic supplementary material

Below is the link to the electronic supplementary material.

**Supplementary Material 1:** The original full-length blot images for Figure 1B and Figure 3B

**Supplementary Material 2: Figure S1**. Silica and alumina oxide particles synergize with influenza viruses to enhance cell death rates at 48 h post-infection. MTS assay evaluating the viability of A549 cells treated with 1, 3, 10, 30, 100 μg/mL PBS, iron oxide, silica oxide, or alumina combined with vehicle (A) AF or (B) H1N1 (M.O.I., 3) or (C) H5N1 (M.O.I., 0.3) virus 48 h post-infection

**Supplementary Material 3: Figure S2**. Silica and alumina oxide particles synergize with influenza viruses to enhance viral loads at different infection stages. (A) q-PCR detection of the influenza virus M2 gene in A549 cells infected with H1N1 (M.O.I., 3) or H5N1 (M.O.I., 0.3) virus combined with PBS, iron oxide (100 μg/mL), silica oxide (100 μg/mL), or alumina (30 μg/mL), separately, at 0.25 h, 0.5 h, 1 h, 2 h, 3 h after infection. (B) q-PCR detection of the influenza virus M1 and M2 gene in A549 cells infected with H1N1 (M.O.I., 3) or H5N1 (M.O.I., 0.3) virus combined with PBS, iron oxide (100 μg/mL), silica oxide (100 μg/mL), or alumina (30 μg/mL), separately, at 6 h, 12 h, 24 h, 36 h, 48 h postinfection. The data are presented as the mean ± S.E.M. of three independent experiments. *P < 0.05, **P < 0.01

## Data Availability

Data sharing does not apply to this article as no datasets were generated or analyzed during the current study.

## List of abbreviations

AF: Allantoic fluid
LAMPs: Lysosomal associated membrane proteins
NA: Neuraminidase
PM: Particulate matter
RT: Room temperature
SDS-PAGE: SDS polyacrylamide gel electrophoresis
TCID50: Tissue culture infectious dose
