# Supplementary material for: Ambient atmospheric PM worsens mouse lung injury induced by influenza A virus through lysosomal dysfunction
Source: Respir Res. 2023 Dec 6;24:306. doi: 10.1186/s12931-023-02618-9 (PMC10699052; doi:10.1186/s12931-023-02618-9)
Supplement: Supplementary file 1 — Supplementary Material 1: The original full-length blot images for Figure 1B and Figure 3B [file 12931_2023_2618_MOESM1_ESM.docx]

**Fig 1B in manuscript：**


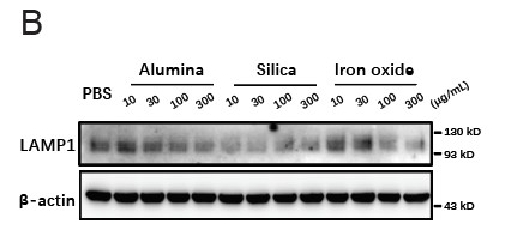


**Original images for Fig 1B：**

**Note:** The two membrane strips in red frame are from the same large membrane, named “A”, which was cut into two strips along the bottom edge of the 72kD marker to save antibodies.

The two pictures in blue frames are the same membrane exposed at different times. As the LAMP1 in the short time exposure was fuzzy, we exposed a longer time to get a clear LAMP1. Finally, we selected the β-actin in short time exposure and the LAMP1 in long time exposure for the figure 1B.


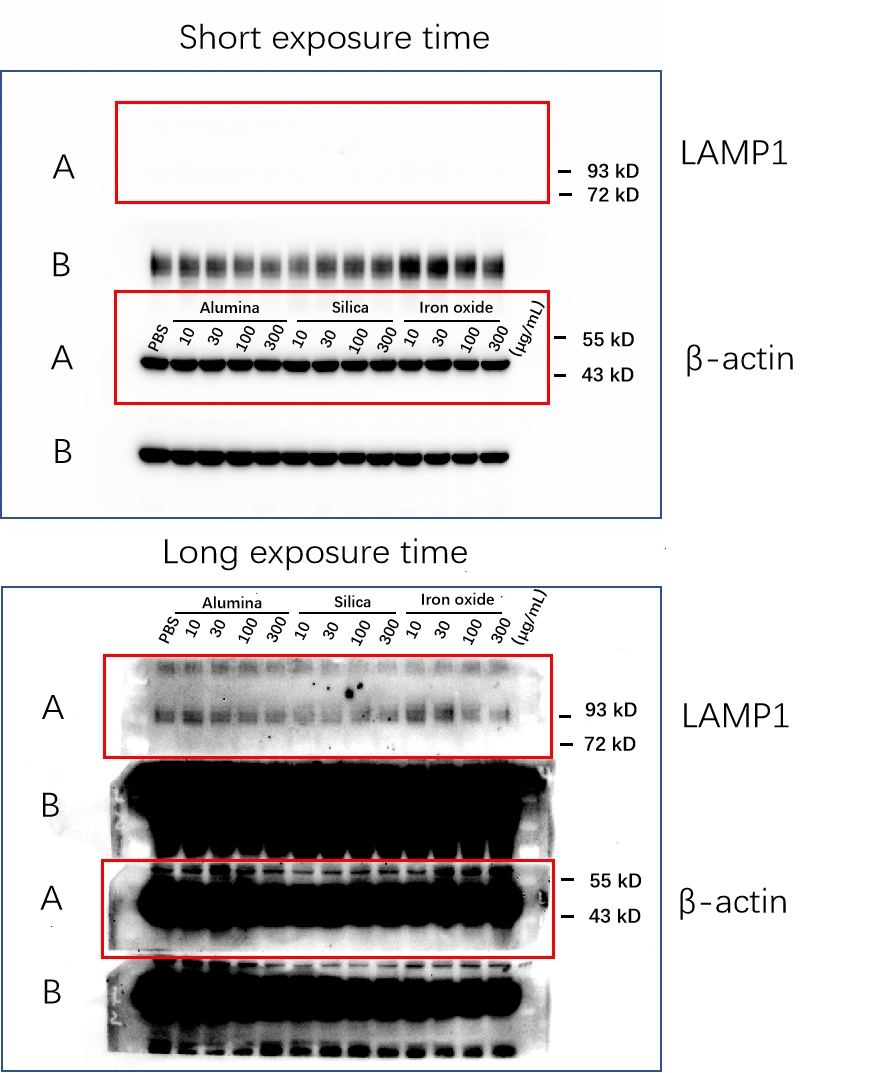


**Figure 1. Lysosomes dysfunction upon incubation with nanoparticles.** (A) A549 cells were incubated with PBS, iron oxide, silica, or alumina for 48 hours, and then their viability was examined by an MTS assay**. (B) Immunoblot analysis of LAMP1 in A549 cells treated with nanoparticles at the indicated concentrations; β-actin was used as the control.** (C) Twenty-four hours post incubation with 30 μg/mL nanoparticles, cells were stained with LysoTracker. Images were acquired using confocal microscopy, and the fluorescence signal intensity of each cell, as shown on the right, was estimated by examining more than 100 cells. The data are presented as the mean ± S.E.M. of three independent experiments. **P* < 0.05, ***P* < 0.01.

**Fig 3B in manuscript：**


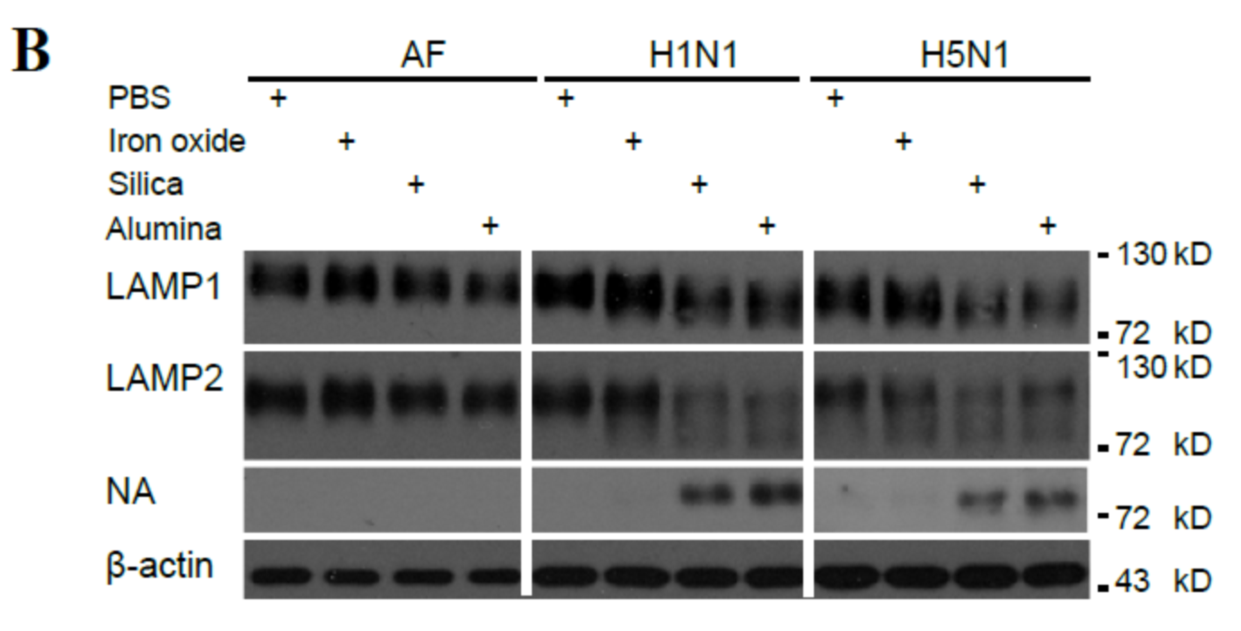


**Original images for Fig 3B**


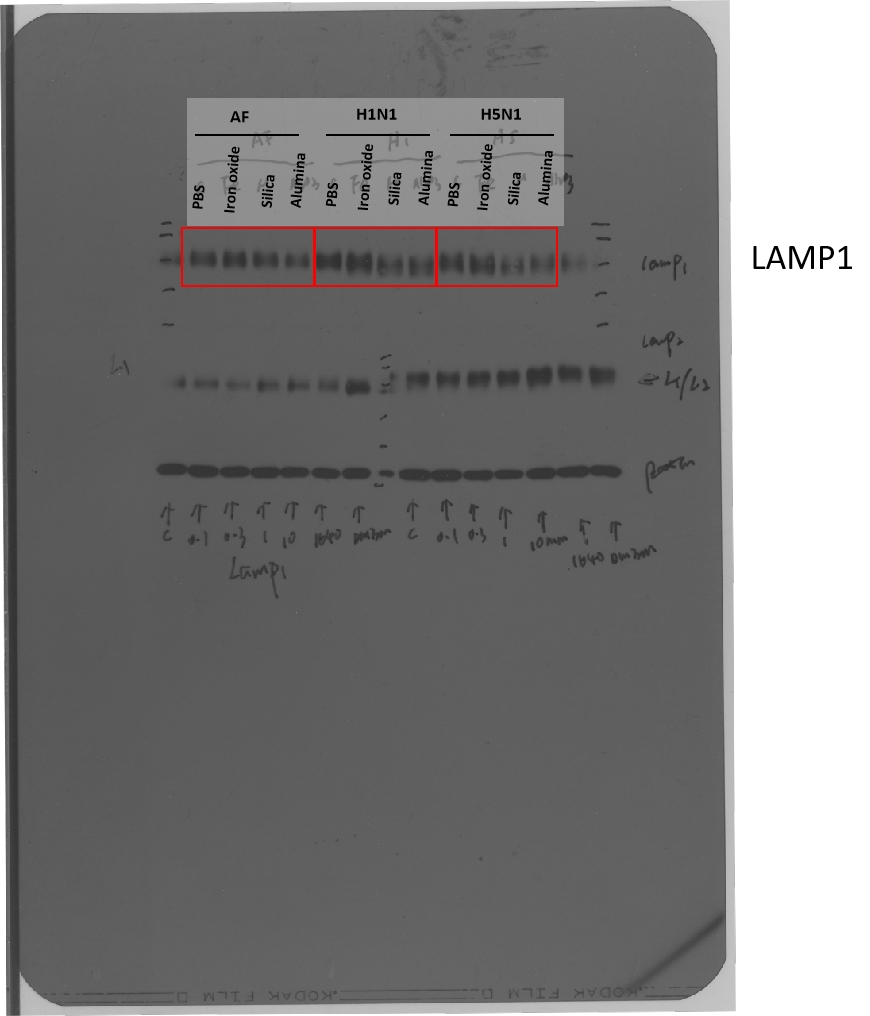


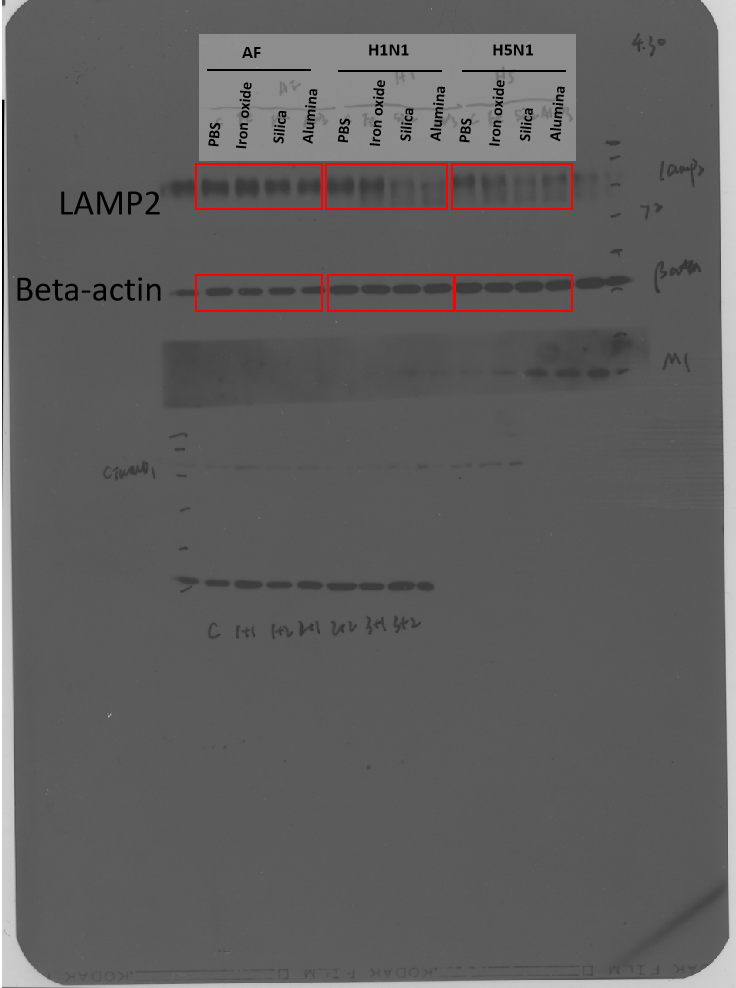


**Note:** The two pictures in blue frames are the same membrane exposed at different times. As the NA of H5N1 group in short time exposure was fuzzy, we exposed a longer time to get it clear. Finally, we selected the NA of AF and H1N1 groups in short time exposure and the NA of H5N1 group in long time exposure for the figure 3B.


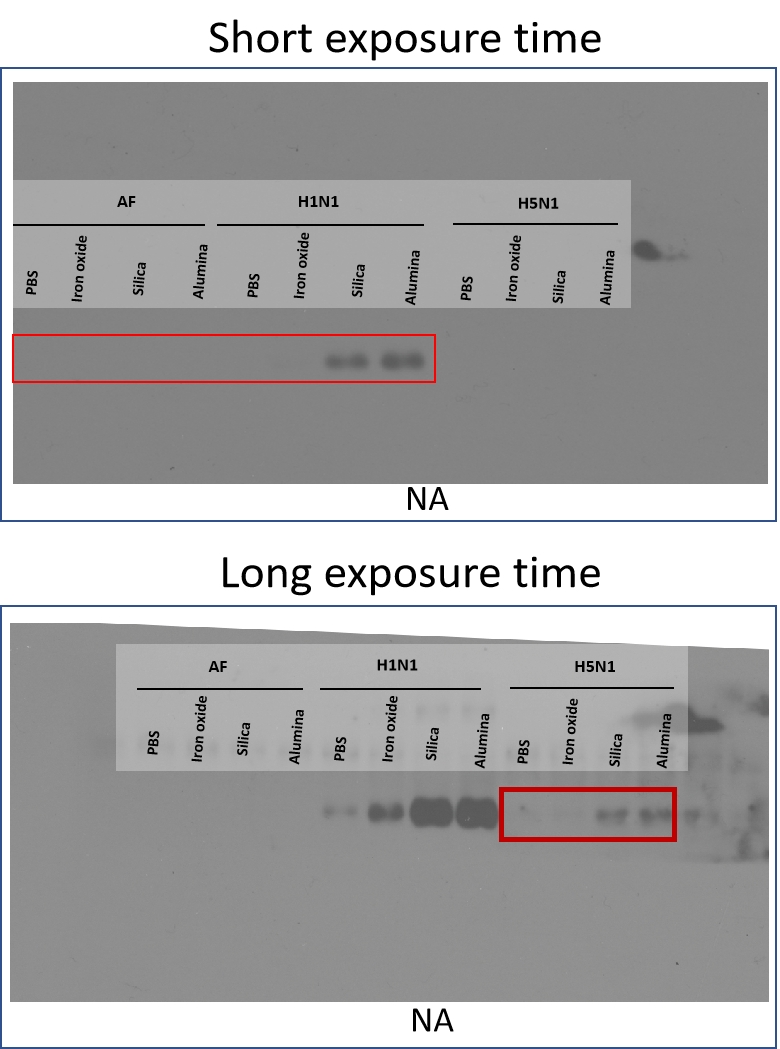


**Figure 3. Silica and alumina oxide particles synergize with influenza viruses to aggravate lysosomal dysfunction.** (A) MTS assay of evaluating the viability of A549 cells treated with PBS, iron oxide (100 μg/mL), silica oxide (100 μg/mL), or alumina (30 μg/mL) combined with vehicle or the indicated amounts of seasonal H1N1 or H5N1 virus 48 hours post-infection. **(B) Immunoblot analysis of LAMP1 and LAMP2 deglycosylation in A549 cells treated with vehicle, H1N1 (M.O.I., 3) or H5N1 (M.O.I., 0.3) virus combined with PBS, iron oxide (100 μg/mL), silica oxide (100 μg/mL), or alumina (30 μg/mL), separately, at 24 hours post infection. LAMP1 and LAMP2 levels were normalized to β-actin levels.** (C) Confocal microscopy analysis of viral NP-positive nuclei in A549 cells treated with vehicle or H1N1 (M.O.I., 3) or H5N1 (M.O.I., 0.3) virus combined with PBS, iron oxide (100 μg/mL), silica oxide (100 μg/mL), or alumina (30 μg/mL) for 4 hours. The graph on the right indicates the percentage of NP-positive cells determined using ImageJ software from at least 1,000 cells. Scale bars, 50 μm. (D) q-PCR detection of the influenza virus M1 gene in A549 cells infected with H1N1 (M.O.I., 3) or H5N1 (M.O.I., 0.3) virus combined with PBS, iron oxide (100 μg/mL), silica oxide (100 μg/mL), or alumina (30 μg/mL), separately, at 0.25h, 0.5h, 1h, 2h, 3h after infection. The data are presented as the mean ± S.E.M. of three independent experiments. **P* < 0.05, ***P* < 0.01.
