# Supplementary figures and images for: Ambient atmospheric PM worsens mouse lung injury induced by influenza A virus through lysosomal dysfunction
Source: Respir Res. 2023 Dec 6;24:306. doi: 10.1186/s12931-023-02618-9 (PMC10699052; doi:10.1186/s12931-023-02618-9)

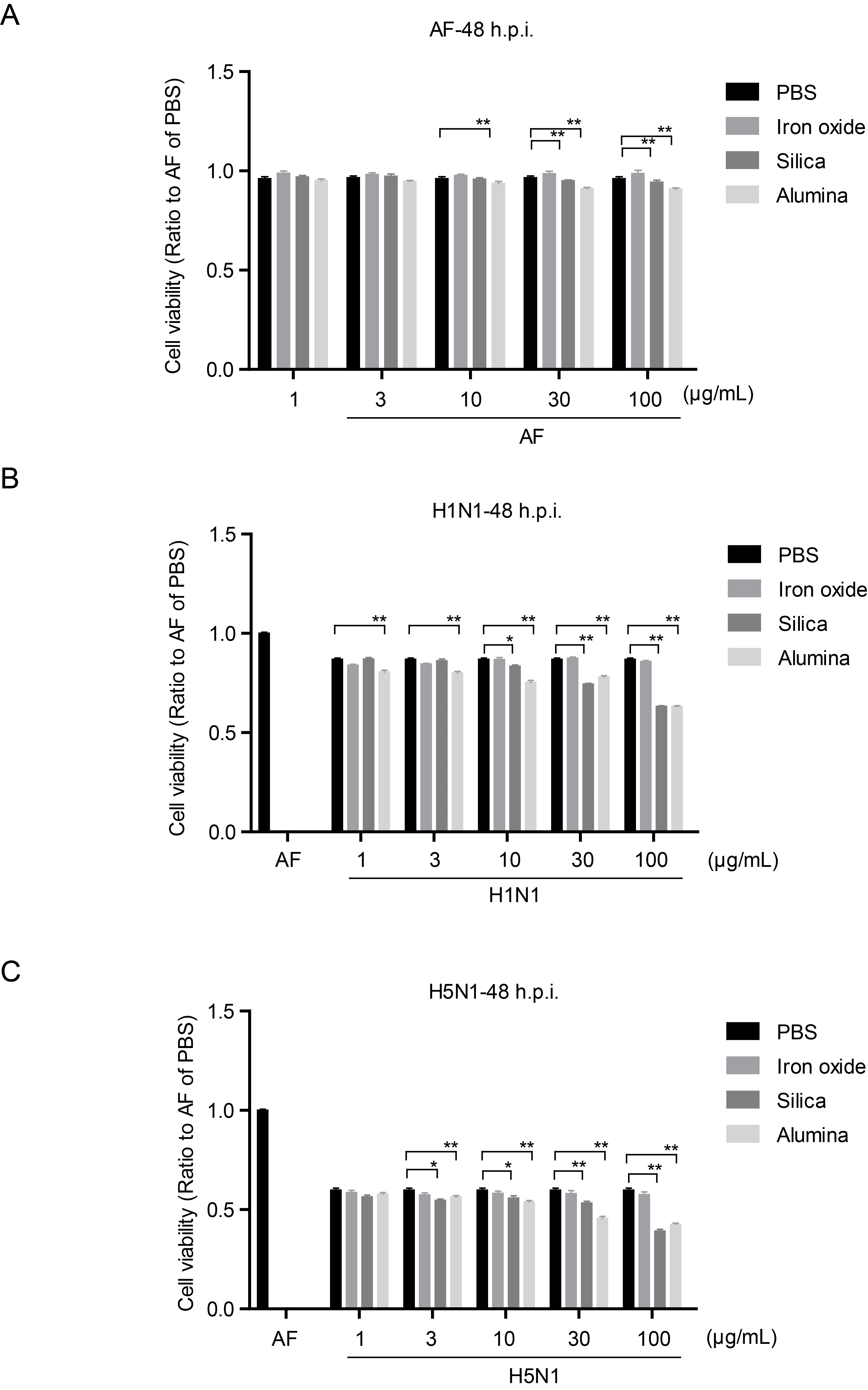

Supplement: Supplementary file 2 — Supplementary Material 2: Figure S1. Silica and alumina oxide particles synergize with influenza viruses to enhance cell death rates at 48 h post-infection. MTS assay evaluating the viability of A549 cells treated with 1, 3, 10, 30, 100 μg/mL PBS, iron oxide, silica oxide, or alumina combined with vehicle (A) AF or (B) H1N1 (M.O.I., 3) or (C) H5N1 (M.O.I., 0.3) virus 48 h post-infection [file 12931_2023_2618_MOESM2_ESM.jpg]

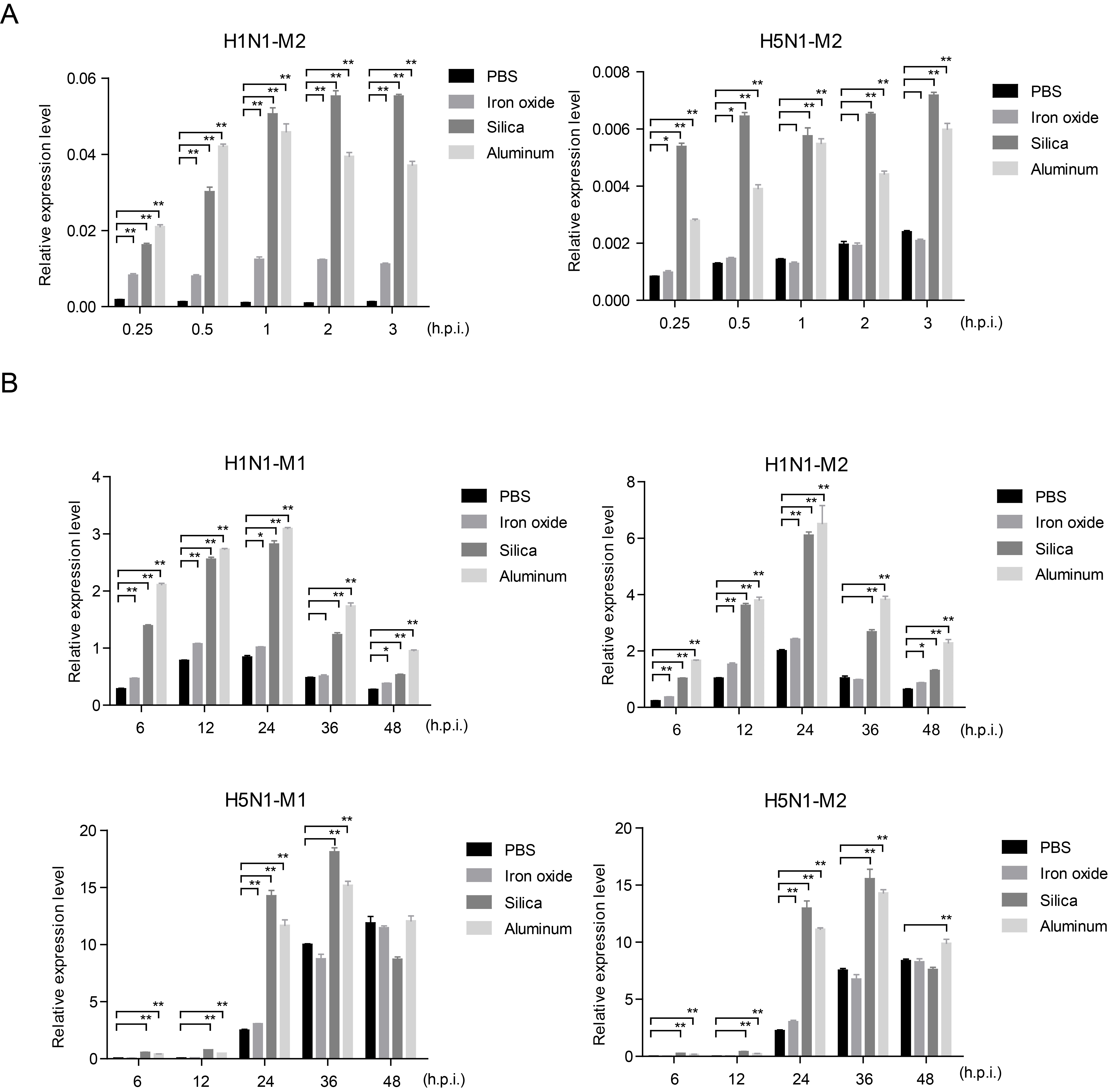

Supplement: Supplementary file 3 — Supplementary Material 3: Figure S2. Silica and alumina oxide particles synergize with influenza viruses to enhance viral loads at different infection stages. (A) q-PCR detection of the influenza virus M2 gene in A549 cells infected with H1N1 (M.O.I., 3) or H5N1 (M.O.I., 0.3) virus combined with PBS, iron oxide (100 μg/mL), silica oxide (100 μg/mL), or alumina (30 μg/mL), separately, at 0.25 h, 0.5 h, 1 h, 2 h, 3 h after infection. (B) q-PCR detection of the influenza virus M1 and M2 gene in A549 cells infected with H1N1 (M.O.I., 3) or H5N1 (M.O.I., 0.3) virus combined with PBS, iron oxide (100 μg/mL), silica oxide (100 μg/mL), or alumina (30 μg/mL), separately, at 6 h, 12 h, 24 h, 36 h, 48 h postinfection. The data are presented as the mean ± S.E.M. of three independent experiments. *P < 0.05, **P < 0.01 [file 12931_2023_2618_MOESM3_ESM.jpg]
